# RETRACTED: Mancinelli et al. The Effects of Taurocholic Acid on Biliary Damage and Liver Fibrosis Are Mediated by Calcitonin-Gene-Related Peptide Signaling. *Cells* 2022, *11*, 1591

**DOI:** 10.3390/cells15020180

**Published:** 2026-01-19

**Authors:** Romina Mancinelli, Ludovica Ceci, Lindsey Kennedy, Heather Francis, Vik Meadows, Lixian Chen, Guido Carpino, Konstantina Kyritsi, Nan Wu, Tianhao Zhou, Keisaku Sato, Luigi Pannarale, Shannon Glaser, Sanjukta Chakraborty, Gianfranco Alpini, Eugenio Gaudio, Paolo Onori, Antonio Franchitto

**Affiliations:** 1 Department of Anatomical, Histological, Forensic Medicine and Orthopedics Sciences, Sapienza University of Rome, 00161 Rome, Italy; romina.mancinelli@uniroma1.it (R.M.); luigi.pannarale@uniroma1.it (L.P.); eugenio.gaudio@uniroma1.it (E.G.); paolo.onori@uniroma1.it (P.O.); 2 Division of Gastroenterology and Hepatology, Department of Medicine, Indiana University School of Medicine, Indianapolis, IN 46202, USA; lceci@iu.edu (L.C.); linkenn@iu.edu (L.K.); heafranc@iu.edu (H.F.); vikmead@iu.edu (V.M.); chenlix@iu.edu (L.C.); kkyritsi@iu.edu (K.K.); nawu@iu.edu (N.W.); zhouv@iu.edu (T.Z.); keisato@iu.edu (K.S.); galpini@iu.edu (G.A.); 3 Richard L. Roudebush VA Medical Center, Indianapolis, IN 46202, USA; 4 Department of Movement, Human and Health Sciences, University of Rome “Foro Italico”, 00135 Rome, Italy; guido.carpino@uniroma4.it; 5 Department of Medical Physiology, Texas A&M University, Bryan, TX 77807, USA; sglaser@tamu.edu (S.G.); schakraborty@tamu.edu (S.C.)

The journal retracts the article titled “The Effects of Taurocholic Acid on Biliary Damage and Liver Fibrosis Are Mediated by Calcitonin-Gene-Related Peptide Signaling” [1], cited above.

Following publication, concerns were brought to the attention of the Editorial Office regarding a range of image irregularities contained within this article [1].

Adhering to our standard procedure, the Editorial Office and Editorial Board conducted an investigation that confirmed a partial overlap of panels presented in Figure 2 and duplicate panels presented in Figure 5B and in Figure 8, showing different experimental conditions. While the authors fully cooperated with the Editorial Office, they were not able to satisfactorily explain the presence of the above-mentioned duplications, nor provide appropriate raw material for Editorial Board evaluation. Consequently, the Editorial Board has lost confidence in the reliability of the findings and decided to retract this publication [1], as per MDPI’s retraction policy (https://www.mdpi.com/ethics#_bookmark30).

This retraction was approved by the Editor-in-Chief of the *Cells* journal.

Romina Mancinelli, Ludovica Ceci, Eugenio Gaudio and Antonio Franchitto disagreed with this retraction. The remaining authors did not provide a comment on this decision.
